# Pandemic Recipes—Nutritional Values of Recipes in Legacy Media

**DOI:** 10.3390/nu17111830

**Published:** 2025-05-28

**Authors:** Ann Gaba, Richard Bennett, Karen Florez, Ghada Soliman

**Affiliations:** Department of Environmental Occupational and Geospatial Health Sciences, Graduate School of Public Health and Health Policy, City University of New York, New York, NY 10027, USA; richard.bennett17@login.cuny.edu (R.B.); karen.florez@sph.cuny.edu (K.F.); ghada.soliman@sph.cuny.edu (G.S.)

**Keywords:** nutrient analysis, legacy media, COVID-19, recipes, descriptions, spices

## Abstract

Background/Objectives: Legacy media are those that existed prior to the advent of the internet. Legacy media have a focus on the needs of specific readership populations. Because of this focus, they remain a trusted source of information for many people. During the COVID-19 pandemic, these media addressed readers’ needs for recipes for home food preparation. Methods: To evaluate the accuracy of the recipe descriptions and to compare these to their estimated nutritional value, we examined 182 recipes extracted from 942 food- and nutrition-related articles in a sample of magazines collected from January to December 2020. Because herbs and spices enhance the palatability of foods, are associated with healthier diet patterns, and provide phytochemicals which may have health benefits, we also examined the inclusion of these in the recipes when comparing their nutritional value. Results: Nutrient comparisons across these groups showed that recipes identified as healthy were the highest in vitamin C, fiber, and potassium and the lowest in cholesterol (*p* < 0.01). Cocktails were about half the calories per serving as all other recipes, and they were substantially lower in all the nutrients evaluated (*p* < 0.01) except for sugars (*p* = NS). An increase in seasonings was associated with higher levels of vitamin A, vitamin C, calcium, potassium, iron, and fiber (*p* < 0.001). Recipes with added seasonings were also lower in sugar (*p* < 0.05). Conclusions: Overall, these results support the hypothesis that recipes in legacy media identified as healthy, as well as those that contained more herbs and spices, were more likely to correspond to healthy diet guidelines.

## 1. Introduction

### 1.1. Legacy Media as a Source of Food and Nutrition Information During the Pandemic

Publications such as newspapers and magazines that have been publishing prior to the advent of the internet are known as legacy media. There has been a significant decline in readership in these publications in print form, but many have reestablished themselves through websites for subscribers in tandem with their print issues [1]. Since this web content is behind a paywall, with limited or no access for non-subscribers, readers must place an economic value on the information provided. In order to retain their subscribers, editors must remain attentive to their interests and needs. Reading level, vocabulary choices, length of articles, tone, as well as specific content are all taken into consideration. Content curation by editors differs from content appearing in social media, which is generally more open-ended, with little or no analysis of the content posted [2,3].

Legacy media have been described as trusted sources of information within specific readership groups [4]. Older readers, as well as readers from specific communities and identity groups, have been identified as being the most likely to seek communications tailored to their specific needs and preferences, and there is evidence that these are the most likely to be impactful in reaching them [5,6,7,8]. It stands to reason that people would turn to their familiar publications for assistance in making food decisions during a global health crisis. Statistics from media industry analyses for 2020–2021 showed an increase in magazine readership during that year [9].

### 1.2. Meeting Specific Physical and Mental Health Needs

It is well known that foods and beverages meet human needs beyond providing nutrients [10]. Foods are associated with meeting a variety of social and emotional needs, along with specific personal health needs [11,12,13]. Descriptions of recipes typically provide descriptions of the product that explicitly state, or at least allude to, the other functions of that recipe. Some examples include comfort foods, celebratory or community foods, foods to promote health, and foods that are quick and easy to prepare. Herbs, spices, and other seasonings also play a role in meeting these needs [14].

Herbs and spices not only provide culinary elements to foods and beverages; they are also potentially protective against acute and chronic diseases [15]. They are a key feature of multiple traditional diet patterns that have been associated with better health outcomes. Some examples include the Mediterranean, Nordic, and Okinawan diets [16] and traditional diets in Sub-Saharan Africa [17] and Thailand [18].

The use of herbs and spices can enhance flavor and decrease salt in the diet, and they have the potential to improve cardiometabolic health [19,20]. They have been shown to provide key nutrients and phytonutrients in human diets [21]. Independently of their specific nutrient contributions, they have been shown to encourage the intake of healthier foods like vegetables in school children [22,23], military service members [24], and people with type 2 diabetes [25].

### 1.3. Pandemic-Related Changes to Diets and Eating Behavior

A systematic review by González-Monroy et al. examined 23 studies from multiple countries looking at changes in eating behavior before and after the beginning of the COVID-19 pandemic [26]. While results differed across the studies they examined, the overall results showed an increased consumption of processed snack foods and sweets, with fewer fresh fruits and vegetables. Additionally, overweight, obese, and people with diabetes reported an increased consumption of starchy foods and sweets. Some groups reported a significant increase in the consumption of alcoholic beverages, with an overall reduction in total food consumption.

Another systematic review examined studies of food intake, eating behaviors, and diet quality before and during the pandemic lockdowns [27]. Most studies were web-based surveys, cataloging self-reported changes in eating behaviors and food consumption patterns, with fifteen studies including an analysis of diet quality. While there was a trend toward healthier diets worldwide, the results were mixed and varied significantly by country and region.

Disruptions of the food supply chain and social distancing requirements changed consumers’ shopping behaviors as well as their food choices [28]. This was a global phenomenon, with food-at-home consumption increasing proportionally to decreased food-away-from-home. United States Department of Agriculture (USDA) data also indicated increased online food purchasing [29]. Increased online food purchasing during this time was also documented in Brazil, with a preponderance of online food vendors advertising less-healthy foods [30]. Lastly, Grunert et al. discussed the impact of the level of trust in the communications received on food behaviors during times of crisis [31]. Lower levels of trust lead to self-guided coping strategies, such as food hoarding due to perceived food insecurity.

### 1.4. Psychological Impact of the Pandemic on Eating Behaviors

In addition to changes in food availability and food acquisition, the psychological impact of a global health crisis and the interventions to mitigate that, such as stay-at-home orders, drove a range of psychological responses in both healthier and less-healthy food behaviors. For some, the pandemic’s disruption of their routines and the fear of illness were drivers of an increased focus on nutrition and health, resulting in improved diets overall [32,33]. Other studies found less-healthy changes, with increases in total energy intake and the intake of sugar, sweets and alcoholic beverages across a variety of populations, including in Brazil [34], Italy [35], New York City [36], the United Kingdom (UK) [37], and the United States (US) [38]. Some of these found a split between genders, with women consuming more energy and sweets, and men consuming more alcoholic beverages [36,38].

An online survey across sixteen countries, including the United States, found two clusters of responses centering on the increased consumption of healthier and unhealthier foods [39]. They note that self-reported mood and emotional states are drivers of food intake, with depression and sadness being strongly associated with less-nutritious choices. As in previous studies, women were more likely to eat less-healthy foods in response to emotional stress. A behavioral survey in the US showed that increased COVID-related stress levels produced an increased willingness to make an effort to acquire fast food or sweets but not healthier options [40].

### 1.5. Increased Home Cooking for Better or Worse

Even prior to the COVID-19 pandemic, there has been a thought that cooking at home would lead to people consuming healthier diets. Looking at the National Health and Nutrition Examination Survey (NHANES) data for the US, Wolfson and Bleich found that the frequency of cooking dinner at home was associated with an overall healthier diet [41]. Similar results were found by Mills et al. [42] in the UK, and again by Wolfson et al. [43].

With a greater amount of time available and limited food choices for eating away from home, one of the consequences of the pandemic mitigation efforts was an increase in home cooking. This seems to have resulted in mixed effects both within and between populations. Cummings et al. found that US adults reported cooking more frequently and increasing their consumption of fruits and vegetables while also decreasing their physical activity [38]. These authors note that although the relationship between the reported stress related to COVID-19 and produce consumption was modest, it was the strongest among respondents with obesity. In New Zealand, Gerritsen et al. found an increase in cooking and baking from scratch, along with a shift to less-healthy eating patterns, including increases in salty snacks, sugary drinks, and alcoholic beverages [44].

An online survey in Europe found that 35% of respondents reported more enjoyment in cooking and eating, including more time in the kitchen and more time spent on family meals [31]. Among those respondents, there was a split between those who were emphasizing healthy choices and those who reported an increased consumption of less-healthy “indulgence food”. Preferences for local products were also noted, which reflected concerns about long-distance supply chains and food safety [39].

### 1.6. Analysis of Recipes for Home Cooks Available Online

While there are many sources of recipes suitable for home cooks available online, there has been little published research examining these. A pre-pandemic study analyzed recipes for home cooks available through the AllRecipes.com website and compared them to recipes in cookbooks from celebrity chefs and ready-to-eat meals available at the three main supermarkets in the UK [45]. Internet-sourced recipes, overall, were less healthy than recipes in cookbooks written by chefs or ready-to-eat meals from supermarkets.

Looking at recipes from AllRecipes.com in the context of the pandemic, Eftimova et al. used an artificial intelligence (AI) method to compare recipes posted pre- and post-pandemic under the categories Diet and Health, World Cuisine, and Cooking Style [46]. To analyze pandemic-era recipes, they included the category Quarantine Cooking. Their findings showed an increase in recipes for foods that could be categorized as “comfort foods” and a decline in recipes for whole grains and seafood. Although they did not perform any nutrient analysis on these recipes, they speculate that these changes are likely to have consequences for both human and environmental health.

### 1.7. Study Hypotheses

The study reported here aligns with the concepts described above by examining the nutritional values of recipes presented in a sample of legacy media published in 2020, the first year of the COVID-19 pandemic. Available recipes were extracted and identified by descriptive identification terms and then compared across these categories by their calculated nutrient content. In addition to this, the number and types of seasonings (herbs, spices, and blends) added to each recipe were tallied and compared by nutrient content. Subsequent analyses were conducted to test the following hypotheses:

**H1.**  *The stated purpose of the recipe will be related to the nutrient density of the product, e.g., foods identified as “healthy” will have a higher nutrient density than foods identified as “quick and easy” or “comfort foods”.*

**H2.**  *Foods containing more herbs and spices will have a higher nutrient density compared with those that do not contain these*.

## 2. Materials and Methods

Data for the present study were extracted for secondary analysis from a database of public health content in legacy media that has been described in detail elsewhere [47]. The original database was created from public health content extracted from eleven periodicals published in English that were publicly available for purchase on newsstands in New York City in November 2019. Inclusion criteria for the database were the following: publication in English, containing at least two public-health-related items in the issue screened, and publication in print as well as having a web-based version available for subscribers.

Eleven magazines, published in English, were included in this original data set, targeting a range of diverse readership groups. These were *The Advocate*, *Elle*, *Essence*, *Gentlemen’s Quarterly* (*GQ*), *Men’s Journal*, *The Oprah Magazine*, (*O*), *Ms. Magazine*, *Out*, *Scientific American*, *Time*, and *Vogue*. One-year subscriptions were purchased for each of these, and relevant material published in print and web-based issues was collected from January through to December of 2020.

### 2.1. Data Extraction

**Figure 1:**
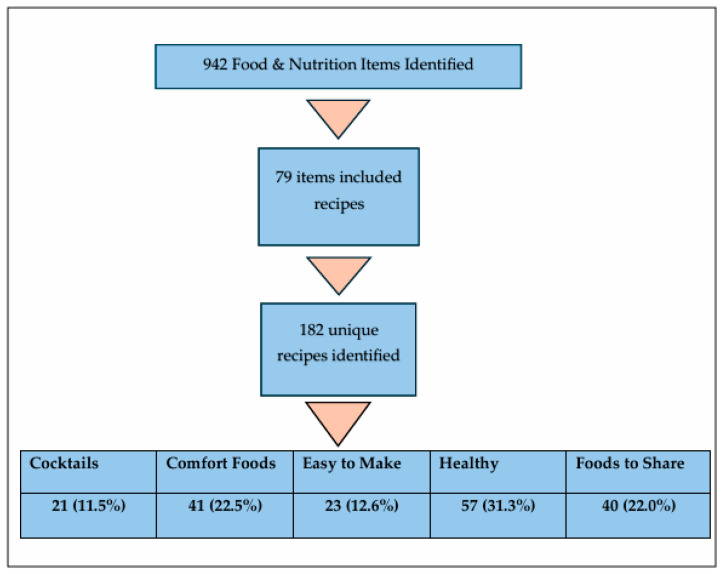
Data extraction process. Flow chart of the process of data extraction from the original data set to the grouping of similar recipes.

The data extraction process is shown in Figure 1 below. A total of 942 food- and nutrition-related items were extracted from the original source materials. There were 370 items found in the print issues and 572 items found on the websites. After removal of duplicates, there were 79 items including recipes: 76 articles and 3 advertisements. Some of these articles included more than one recipe. Each recipe was coded separately, with a total of 182 unique recipes.

All food- and nutrition-related articles from the original data set were examined to identify those containing recipes. Three magazines, *Ms.*, *GQ*, and *Scientific American*, had included nutritional content but did not include recipes. This was determined prospectively during our analysis, which then resulted in those three magazines being removed from further analysis. Thereafter, the eight periodicals included in the recipe analysis were the *Advocate*, *Elle*, *Essence*, *Men’s Journal*, *O* (*The Oprah Magazine*), *Out*, *Time*, and *Vogue*. Since this secondary analysis was entirely focused on recipes, it is unlikely that the excluded magazines contributed to bias in the results.

### 2.2. Content Analysis

Content analysis is a method for categorizing and examining objective and subjective ideas and themes embedded in materials such as open-ended survey questions, transcripts of interviews or videos, and other texts. It has a long history of use in the social sciences [48] and also in health professions [49,50,51]. Bengtsson provides a concise overview of the content analysis methodology [52].

Through an inductive content analysis approach, theme codes were created as the collected documents were reviewed. To examine recipes embedded within this data set, articles and advertisements containing recipes were extracted from the larger data set. A set of codes specific to categorizing recipes was created to include the type of recipe, function or intention of the recipe, and the inclusion of herbs, spices, and other seasonings.

The development of codes for botanical products has been described in detail elsewhere [53]. The same coding methods were used here. New codes were added until no new items were identified. Both authors examined the coded data in turn. Where discrepancies were noted, these were discussed until a point of consensus was reached.

### 2.3. Data Coding for Recipes

All the data assigned to each document containing one or more recipes were extracted and copied to an Excel file. The titles given to each recipe were then added to each of these files. When a document contained more than one recipe, a separate file was created for each recipe including all prior identifiers, with an additional code for article number 1 through to n as a unique identifier for that recipe within each given document. Each recipe was then classified according to two different criteria: the type of recipe and the function of the recipe.

The type of recipe referred to the meal or portion of a meal that the product of that recipe was indicated as being. The codes developed for the type of recipe included beverages, appetizers, breads, breakfast foods, entrees, side dishes, desserts, cookies, snack foods, and other foods.

Recipe groupings were created during the data extraction process. These were based on the predominant descriptive wording in the title and surrounding text for each recipe. From examining the recipes and the descriptive terms in each of them, there were five clusters of recipe types that included similar terminology. Cocktails were mixed drinks containing one or more forms of alcoholic beverages. Comfort foods were identified by the phrase “comfort food” or similar words such as “cozy meal”, “for a stressful time”, or “pamper yourself with”. Easy-to-make recipes were items that either had that exact phrase in the title or description or similar phrasing, such as “quick and easy”, or “simple to make”. Healthy recipes were defined by the use of the word “healthy” in the title or description or by similar terms such as “health supporting” or “good for you”. Foods to share were recipes producing a larger number of servings, with descriptive terms such as “family-style”, “community meal” or “for your gathering”. These categories were intentionally kept rather broad to minimize the subjectivity inherent in creating categories based on commonalities in text-based materials. Where there was any ambiguity, the wording in the recipe title was prioritized over the descriptive text.

Recipes that did not fit into any of these categories were coded as “other”. Recipes that fell into this category were for items that would not be eaten on their own, such as dipping sauces, toppings, or flavored oils that were not included as a part of any other type of recipe.

### 2.4. Descriptive Statistics

**Figure 2:**
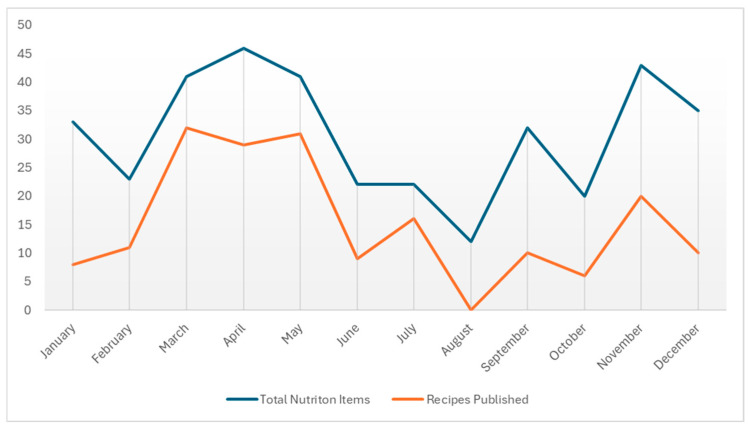
Total number of food and nutrition items by month vs. total number of recipes by month. Timeline of the total number of nutrition items published per month through 2020 compared with the total number of recipes published.

The food and nutrition content and the number of recipes were not distributed evenly throughout the year. The number of food and nutrition items over each month of 2020, along with the total number of recipes per month are shown in Figure 2. It is worth noting that both of these increased sharply in March at the outset of the COVID-19 pandemic. This potential relationship and its consequences are discussed further below.

**Figure 3:**
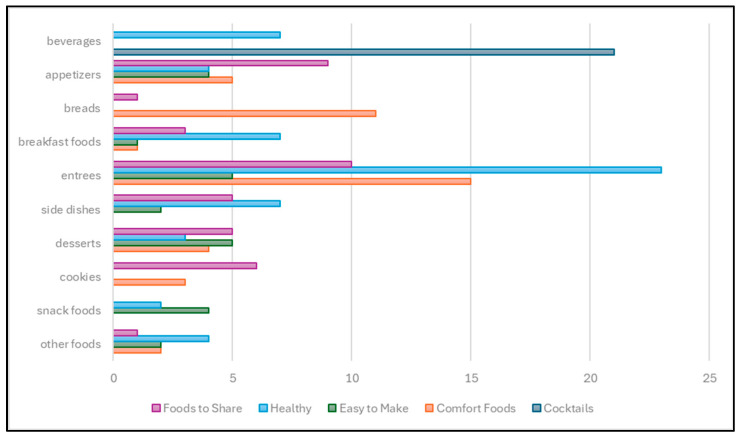
Type of food by function of recipe. Comparison of the types of foods produced with the descriptive functions within the recipes.

Figure 3 shows the types of foods produced by the recipes as compared to the primary descriptive term for that recipe. Healthy foods was the most prevalent category, with entrees representing the largest group within that category. Comfort foods and foods to share had comparable totals, but comfort foods included a higher number of recipes for breads, with foods to share being more evenly distributed across the types of food.

### 2.5. Types of Recipes and Functions of Recipes in Different Magazines

There was a high degree of variability as to how many recipes were included in each magazine and the types of recipes they each featured. *Men’s Journal*, *Oprah*, and *Vogue* had the highest total numbers of recipes included and the highest number of entrees, while *Essence*, *Time*, and *The Advocate* had the fewest total recipes. The *Oprah* magazine included the highest number of appetizer, dessert, cookie, and bread recipes and the greatest variety of recipes across categories, with *Vogue* having lower totals but a similar variety distribution.

The functions of the recipes also varied by periodical source. Healthy recipes was the largest overall category, with *Men’s Journal*, *Oprah*, and *Vogue* contributing the most. *Out* and *Men’s Journal* included the highest number of cocktail recipes. *Vogue* and *Oprah* included the most comfort food recipes, with *Oprah* also having, by far, the most easy-to-make recipes. Recipes for foods to share were found in highest numbers in *Men’s Journal* and *Oprah*.

**Figure 4:**
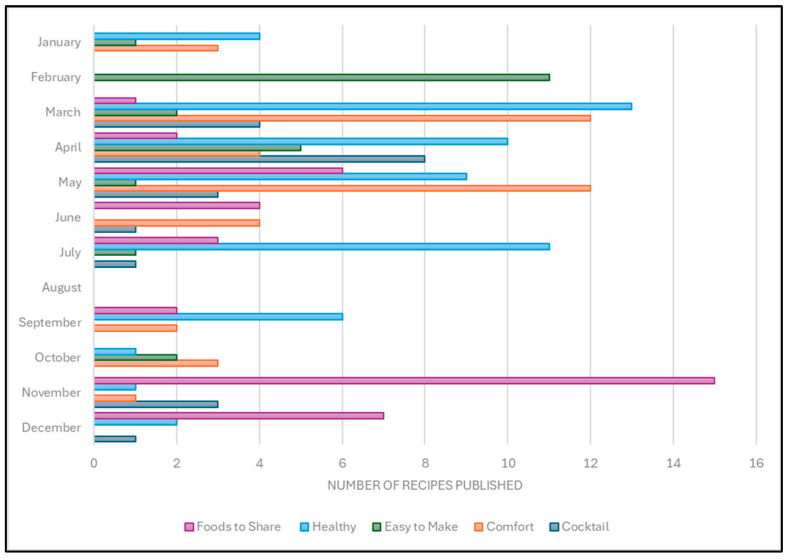
Function of recipe by month of publication. Number of recipes by each descriptive function published each month through 2020.

The function of the recipes provides a different description of what kinds of products were the most prevalent each month. These are shown in Figure 4. Easy-to-make recipes reached a maximum in February. Recipes for both comfort foods and healthy foods peaked in March. Recipes for cocktails reached their maximum inclusion in April. By November, foods-to-share became the most prevalent category, consistent with the winter holidays.

### 2.6. Nutrient Analysis

#### 2.6.1. Description of Software and Database

Analysis of a specific food from a specific source requires laboratory analysis. Comparable data would not be available in any database and so would need to be generated de novo. While determining moisture and ash content in the proximate analysis of food is, in addition to its nutrient content, required in describing new food for quality control and regulatory purposes, an analysis of nutrient content is conducive to comparative food analysis. For example, if nutrient analysis of a new food product is needed for labeling, then that unique food item would require proximate analysis. However, for a more general analysis of common foods, with no reference to a specific source of those foods, nutrient analysis software will generate the best results.

For the study reported here, the Nutritionist Pro nutrient analysis software was chosen as the method for comparative analysis because preparing each of the recipes and carrying out multiple proximate analyses for each nutrient would have been more likely to introduce errors due to food sourcing differences, the regional variability of ingredients, or other causes. The data referenced in Nutritionist Pro are sourced from the well-documented USDA Nutrient Database for Standard Reference, as well as from data provided for branded food products from the manufacturers (Redmond, WA, USA, Axxya Systems, 2024).

The USDA Nutrient Database for Standard Reference is updated annually, and these updates are incorporated into updates to Nutritionist Pro when they become available. The USDA methodology for creating database updates is described by Jaspreet et al. [54]. Extensive metadata for the database source data, including sampling procedures and analytical methods, including all of the citation references to the original laboratory analyses, are available at the USDA Data Commons website [55].

Data from industry sources for specifically branded products are derived from their food labeling, where a high degree of accuracy is required, which is subject to Food and Drug Administration (FDA) legal requirements. These are available from the FDA regulations website [56].

#### 2.6.2. Nutrient Analysis Procedure

Recipes were analyzed using the Nutritionist Pro recipe analysis template. Because of the consistency of analysis provided by this software, we did not need to analyze replicates of each dish. This is one of the advantages of using nutrient analysis software. Each ingredient in each recipe was entered individually in the quantity specified by the recipe. Composite ingredients such as ketchup were entered under the codes for those ingredients. If brand names were specified in the recipe, e.g., Heinz Ketchup, the entry was made using the brand name. If no specific brand was given, the item was entered generically, e.g., ketchup.

The analysis output included the following: total calories per serving; protein; total fat; saturated fat; carbohydrate; sugars; fiber; vitamins A, C, D, and E; and the minerals iron (Fe), calcium (Ca), magnesium (Mg), potassium (K), and sodium (Na).

Descriptive statistics for these variables were computed using Microsoft Excel 365. Comparative statistics were computed using SPSS Version 27, IBM 2020, and RStudio 2023.09.0+463 “Desert Sunflower” 2023.

## 3. Results

### 3.1. Nutrient Analysis—H1: Recipe Descriptions vs. Nutrient Content

Nutrients of interest were identified and were extracted from the overall results. The median values for the selected nutrients were compared to the Dietary Reference Intakes (DRIs) as shown in Table 1. Nearly all values were significantly different from the DRI values (*p* < 0.05), indicating that one typical serving of the foods listed in each category would provide less than the DRI for each nutrient examined.

Exceptions to this were vitamin A in appetizers, breakfast foods, cookies, and snack foods and vitamin C in snack foods, whose values for these recipe categories were similar to the DRI value within a 95% confidence interval. This indicated no significant differences.

To test H1, which was that the stated purpose of the recipe would be related to the nutrient density of the product, e.g., foods identified as “healthy” will have a higher nutrient density than foods identified as “quick and easy” or “comfort foods”, all the recipes were analyzed for their nutrient content utilizing the Nutritionist Pro software (Redmond, WA, USA, Axxya Systems, 2024), as described above.

To make comparisons easier to visualize, the relative differences in nutrient content across the various recipe functions are shown as medians for each nutrient in Table 2. Comparisons of the median values for each nutrient across recipe functions were performed using the U test, as described by Kerby [59]. Recipes identified as healthy were the highest in vitamin C, fiber, and potassium and the lowest in cholesterol (*p* < 0.05). No significant differences were noted for other nutrients. Cocktails were about half the calories per serving as all other recipes and were substantially lower in all nutrients evaluated (*p* < 0.01) except for sugars and vitamin C (*p* = NS). These results support Hypothesis 1.

### 3.2. H2: Analysis of Seasoning Content vs. Nutrient Content

H2 was that foods containing more herbs and spices would have a higher nutrient density compared with those that did not contain these. To test this second hypothesis, the types, varieties, and quantities of herbs and spices and other seasonings in each recipe were quantified and compared by the type of recipe, the purpose of the recipe, and the overall nutritional value.

Across the 182 recipes evaluated, 119 different seasonings were included. Of these, 91 were individual herbs or spices (e.g., cilantro, ginger), with the remainder being blends such as fish sauce or curry powder. Each recipe contained between zero and ten different seasoning ingredients.

Entrees and side dishes had the highest number of seasonings added, while beverages and cookies had the lowest. Quantities ranged from zero to as many as ten per recipe. The average (mean) number of seasonings added was 2.76 per recipe, with a standard deviation (SD) = 2.36 and a variance of 5.55.

Nutrient content (median) per serving compared by the number of seasonings included in each recipe, as seen in Table 3, showed the most substantial differences (*p* < 0.001) for protein, sodium, vitamin A, vitamin C, calcium, fiber, potassium, and iron for recipes including 3–6 added seasonings compared with those with none. Comparing 1–2 added seasonings to 5–6 also showed significant differences for the same nutrients (*p* < 0.001). Comparing recipes with 3–4 and 5–6 seasonings showed significantly higher amounts of fiber, potassium, and iron (*p* < 0.001). When more seasonings were added no significant differences were noted for the other nutrients examined. Additional seasonings beyond 5–6 did not show further increases in the nutrient content. These results support H2 to a limited extent, with diminishing returns found above the 5–6 range (*p* = NS).

## 4. Discussion

### 4.1. Key Findings

#### 4.1.1. Types of Recipes

The COVID-19 lockdowns shifted people’s cooking behaviors toward an increase in home cooking and baking, famously of sourdough and banana bread [60]. Amateur mixologists also took up the challenge of preparing cocktails for Zoom happy-hour events [61]. In addition, many people increased their home cooking of meals for themselves and for others sharing quarantine quarters together. To meet the need for recipes and instructions on how to carry out these preparations, home cooks turned to a variety of resources. Legacy media were early to adapt to these needs in both print and web-based editions.

The number and types of recipes varied over the span of the entire year, with the largest number of recipes published between March and May. There was also an increase in November, as would be expected prior to the winter holidays. It is worth noting that the category of recipes for “beverages” was highest in April, concurrent with ongoing pandemic restrictions in many areas. There was also some differentiation in the types of recipes seen based on sources and primary readership groups. *Out* and *Men’s Journal* included the highest number of cocktail recipes, while *Vogue* and *Oprah* included the most comfort food recipes.

#### 4.1.2. Function of Recipes

While these findings show differences across readership groups as expected, they do not entirely align with gender expectations. As was shown in our findings above, healthy recipes in these legacy media were the largest overall category, with *Men’s Journal*, *Oprah,* and *Vogue* contributing the most. Recipes for foods to share were found in highest numbers in *Men’s Journal* and *Oprah*, whose readership groups are likely to diverge on many other interests.

Our findings supported our first hypothesis, that the stated purpose of each recipe will be related to the nutrient content of the product, e.g., foods identified as “healthy” will have a different nutrient composition than foods identified as “easy to make” or “comfort foods”, which indicates a degree of reliability from the sources of these recipes. This was the case in comparing recipes identified as healthy to all others, as those recipes were higher in vitamin C, fiber, and potassium and the lowest in cholesterol (*p* < 0.05). The healthy recipes were also lower in the average amount of sodium per serving, but the differences were not statistically significant. This may have been due to the use of salt-containing spice blends, which is discussed further below.

#### 4.1.3. Number of Seasonings in Recipes and the Nutrient Content

“The Dietary Guidelines for Americans recommend using spices and herbs to reduce sodium, added sugars, and saturated fat. They also encourage using spices and herbs to make nutrient-dense meals more enjoyable” [62] (p. 27). Our results were to some extent consistent with this guidance.

Increasing the number of seasonings added was consistent with higher levels of vitamin A, vitamin C, calcium, potassium, iron, and fiber (*p* < 0.001) for recipes including 3–6 added seasonings compared those with none. Comparing 1–2 with 5–6 added seasonings also showed significant differences for the same nutrients. (*p* < 0.001). Comparing 3–4 and 5–6 added seasonings showed significantly higher amounts of fiber, potassium, and iron when more seasonings were used (*p* < 0.001). Additional seasonings beyond 5–6 did not show further increases in the nutrient content (*p* = NS).

Recipes adding up to six seasonings were lower in sugar than those with no added seasonings (*p* < 0.05). Adding more than six seasonings made no additional difference.

While the increased use of herbs and spices has been reported to promote reduced sodium intake [19], our results showed increasing amounts of sodium with additional seasonings (*p* < 0.001). This was likely due to the inclusion of seasoning blends that contain salt in our analysis. The use of individual herbs and spices as opposed to salt-containing blends can help in lowering dietary sodium intake as was seen in an interventional study by Okeda et al. [63]. Proximate analysis of seven common spices by Al Dhaheri et al. [64] indicated that spices themselves are not significant sources of sodium. They did, however, identify some spices as sources of calcium and potassium, consistent with our results.

### 4.2. Interpretation

#### 4.2.1. Importance of Accurate Food and Nutrition Information from Media Sources

As people navigated their way through the initial year of the COVID-19 pandemic, food-related behaviors were affected by their own internal factors, such as stress and coping skills, as well as external factors such as food insecurity, supply chain issues impacting food availability, and limitations on the availability of food away from home [65]. They sought information from a variety of media sources to help them address their needs [66]. Comparing different types of media, a study by Bridgman et al. found substantial differences in the accuracy of information about COVID-19 disseminated through traditional news sources and via posts on Twitter [67]. A multinational review of COVID-19 misinformation in media found that social media was responsible for greater than 80% of misinformation, whereas only 3% came from traditional media sources, newspapers, magazines, and television news [68]. Consistent with this, studies in Asia [69] and Norway [70] found a greater degree of trust in traditional media.

In addition to the general influence of media on food intake, whether it increased or decreased, inaccurate information can result in poor choices regardless of individual behavioral intent. For example, a study of recipes posted on Instagram labeled #healthy found that many of these did not align with standard definitions of healthy foods [71].

#### 4.2.2. Directions for Further Research

Further studies of nutrition content in legacy media may include other potential benefits of herbs and spices beyond the scope of the present study. In addition to their relationship to macro and micro-nutrient intake, herbs and spices are high in bioactive compounds, which may have health benefits [72]. Data-mining studies using biomedical literature have shown broad-spectrum health benefits from the consumption of herbs and spices [73], as well as specific activities against known diabetes drug targets [74].

Doses consistent with usual culinary applications have the potential for the prevention or treatment of disorders like metabolic syndrome [75]. Some beneficial phytochemicals that have been identified in herbs and spices include quercetin, curcumin, kaempferol, luteolin, and catechin [76]. These have been identified as having antioxidative and anti-inflammatory properties [77] and potential benefits in the prevention and treatment of cardio-metabolic disorders, autoinflammatory diseases, cancer, and cognitive disorders [76]. Interventional studies of herb and spices in both a single-dose feeding trial [78] and a longer-term RTC [79] showed improvements in lipid and inflammatory markers.

An increased consumption of herbs and spices has been associated with adherence to healthier diet patterns. Many of these seasonings are found in the Mediterranean diet [80] and are associated with improved cardiometabolic health [81]. Other traditional diets, such as the Nordic and Okinawan diets, and the Dietary Approaches to Stop Hypertension (DASH) diet also include significant amounts of herbs and spices [82].

#### 4.2.3. Strengths of This Study

Analysis of legacy media is not often conducted, even though significant numbers of people still utilize these as a source of trusted information. This was particularly important when pandemic restrictions made it necessary for people to cook more at home. Less-experienced cooks require not only access to appealing recipes with clear directions, but accurate nutrition information, whether implicit or explicit, to support their diet choices.

Our results showed that recipes identified as being healthy were, in fact, higher in several nutrients, as well as being lower in cholesterol. Higher levels of seasoning were also associated with higher levels of some nutrients and, importantly, with lower amounts of sugar.

Examination of recipes for cocktails showed lower levels of nutrients and more sugar per serving than other foods. While these are not surprising findings, they do provide a degree of quality check on our nutrient analyses of other recipes, thus increasing our confidence in those results.

The pattern of recipes for cocktails, although different from the typical monthly patterns of alcohol consumption in the US [83], was consistent with other literature showing an atypical increase in the consumption of alcoholic beverages in March and April, concurrent with pandemic lockdowns [84,85].

#### 4.2.4. Limitations of This Study

The primary limitations of this study are the limited sample of magazines reviewed, that the magazines were restricted to those in English, and the time-bound nature of legacy media. Although the magazines sampled in our study were chosen to represent a wide range of readership groups, they may not have been representative of all legacy media in the United States or the world. Furthermore, data from 2020 represent an unusual year. Data collected during this unique point in history may have captured the unusually strong influence of the COVID-19 pandemic.

Another limitation was the inability of our analysis to determine with greater precision the nutritional content of the seasonings used in the recipes examined, particularly with regard to sodium. Each recipe contained between zero and ten different seasoning ingredients. Because the recipes contained varying numbers of seasonings, some of which were individual herbs or spices and some of which were mixtures, it was not possible to analyze each one separately in this setting.

Proximate analysis of nutrient content of specific herbs and spices has been reported by others, with the amounts of sodium averaging about 20–40 milligrams per 100 g [86]. Product information from an industry source indicated a wide variability of sodium content in various spice blends, from a trace amount in Shichimi Togarashi seasoning to more than 60 g of salt per 100 g of product in Hickory Smoke flavored salt [87]. Given the available information about the sodium content of individual herbs and spices and the range of sodium in seasoning blends, it is logical to surmise that the differences in sodium that we observed were due to the amount of salt contained in the seasoning mixtures and not the herbs or spices themselves.

## 5. Conclusions

As cooking at home increased during the pandemic, it was important to have accurate information describing the purpose of the recipes people acquired from various media. During this tumultuous time, our study found that recipes identified as healthy in legacy media were, in fact, nutritionally consistent with factors contributing to a healthy diet.

As consumers seek out trustworthy sources of food and nutrition information, it is important to be able to identify accurate materials and to discern the extent of that accuracy. For example, our finding that an increased number of seasonings was not consistent with lower sodium intake points out the need to educate consumers about reading labels to identify seasoning blends containing added salt.

In educating consumers, providing information on what is correct and reliable is just as important as pointing out misinformation. Further research exploring a wider range of legacy media sources could promote an understanding of this important source of nutrition information for the public.

## Figures and Tables

**Table 1:** Nutrient content of recipes compared to Dietary Reference Intakes.

	Vit A ^a^	Vit C ^a^	Vit D ^a^	Vit E ^a^	Fe ^a^	Ca ^a^	Mg ^a^	K ^a^	Na ^b^
DRI	900 μg	90 mg	20 μg	15 mg	18 mg	1300 mg	420 mg	2600 mg	2300 mg
Appetizers	469.05 NS (862.93)	17.044 *** (19.07)	0.024 *** (0.10)	0.00 *** (0.07)	1.80 *** (1.71)	69.81 *** (107.55)	40.07 *** (22.93)	375.14 *** (434.32)	652.55 *** (654.01)
Beverages	119.39 *** (251.01)	8.65 *** (20.20)	0.00 *** (0.00)	0.00 *** (0.00)	0.27 *** (0.66)	10.87 *** (27.60)	7.22 *** (7.69)	81.24 *** (89.58)	14.68 *** (298.47)
Breads	180.99 *** (270.89)	2.04 *** (2.61)	0.08 ** (0.19)	0.09 ** (0.08)	2.10 *** (1.53)	62.59 *** (70.46)	14.69 *** (19.91)	147.48 *** (158.27)	401.51 *** (227.86)
Breakfast foods	273.27 NS (252.79)	8.71 *** (7.20)	0.29 ** (0.92)	0.10 ** (0.27)	2.14 *** (2.30)	148.36 *** (124.43)	62.68 *** (104.12)	585.51 *** (542.46)	527.71 * (1512.49)
Cookies	198.81 NS (41.66)	0.09 ** (0.07)	0.06 ** (0.03)	0.04 ** (0.02)	0.80 ** (0.35)	13.03 ** (25.93)	7.56 ** (12.64)	40.90 ** (93.68)	84.87 ** (163.59)
Desserts	489.86 ** (795.48)	0.31 *** (1.30)	0.25 *** (0.38)	0.09 *** (0.15)	1.65 *** (2.54)	66.64 *** (57.70)	21.48 *** (32.94)	127.42 *** (170.66)	344.94 *** (226.15)
Entrees	1860.22 *** (3190.01)	27.38 *** (48.96)	0.22 *** (0.72)	0.00 *** (0.14)	4.16 *** (2.48)	142.06 *** (143.10)	77.46 *** (61.05)	765.11 ** (420.72)	993.49 *** (959.84)
Side dishes	1628.13 * (913.16)	49.20 * (20.78)	0.00 *** (0.00)	0.00 *** (0.00)	1.94 *** (1.12)	95.36 *** (53.31)	50.53 *** (31.18)	708.35 *** (274.46)	722.72 *** (285.02)
Snack foods	781.63 NS (2343.54)	11.23 NS (51.36)	0.02 * (0.15)	0.00 * (0.04)	2.33 * (1.24)	89.68 * (54.11)	55.55 * (25.28)	390.53 * (359.74)	298.33 * (556.17)
Other	514.16 NS (768.31)	14.63 ** (9.21)	0.00 ** (0.00)	0.00 *** (0.00)	0.69 ** (1.84)	38.22 ** (58.43)	12.81 ** (52.78)	254.06 ** (469.61)	165.31 ** (357.58)

Comparison of median nutrient values (and interquartile range (IQR)) for each recipe type to DRIs. * *p* < 0.05; ** *p* < 0.01; *** *p* < 0.001. ^a^ Institute of Medicine [57]; ^b^ National Academies of Sciences, Engineering, and Medicine [58]. 95% confidence intervals for NS = Vit A—Appetizers [373.32–1091.44]; Vit A—Breakfast Foods [166.54–1880.47]; Vit A—Cookies [109.17–982.01]; Vit A—Snack Foods [77.75–1264.72]; Vit A—Other [101.78–8221.21]; Vit C—Snack Foods [0.31–131.70].

**Table 2:** Comparison of nutrient content of recipes by function.

	Comfort Foods	Cocktail	Easy to Make	Healthy	Foods to Share
Kilocalories	403.36 ^a^ (327.92)	192.46 ^b^ (36.58)	362.79 ^a^ (341.60)	380.02 ^a^ (286.50)	317.56 ^a^ (276.37)
Protein (g)	9.03 ^a^ (11.95)	0.30 ^b^ (0.43)	8.17 ^a^ (13.72)	11.18 ^a^ (13.96)	7.52 ^a^ (11.43)
Carbohydrates (g)	39.42 ^a^ (31.93)	14.25 ^b^ (8.97)	34.39 ^a^ (43.86)	32.80 ^a^ (39.21)	30.71 ^a^ (32.34)
Fat (g)	19.71 ^a^ (18.05)	0.10 ^b^ (0.20)	20.06 ^a^ (26.67)	15.84 ^a^ (19.07)	15.47 ^a^ (19.52)
Sodium (mg)	505.01 ^a^ (1287.26)	7.36 ^b^ (127.36)	403.02 ^a^ (941.46)	630.61 ^a^ (753.99)	674.18 ^a^ (885.46)
Vitamin A (IU)	514.16 ^a^ (1618.16)	107.09 ^b^ (215.06)	653.85 ^a^ (1489.56)	600.54 ^a^ (1072.08)	668.06 ^a^ (1702.90)
Vitamin C (mg)	2.99 ^a^ (28.20)	10.47 ^a^ (19.45)	4.70 ^a^ (18.51)	20.18 ^b^ (49.19)	18.40 ^ab^ (36.27)
Calcium (mg)	129.04 ^a^ (142.55)	9.45 ^b^ (8.26)	98.03 ^a^ (105.89)	100.39 ^a^ (127.45)	82.27 ^a^ (102.19)
Fiber (g)	2.77 ^a^ (5.33)	0.21 ^c^ (0.65)	4.89 ^ab^ (7.01)	5.81 ^b^ (5.47)	2.81 ^a^ (4.35)
Sugar (g)	7.81 (17.14)	11.64 (7.31)	6.67 (20.90)	8.38 (12.43)	9.97 (17.82)
Cholesterol (mg)	31.19 ^a^ (78.43)	0.00 ^b^ (0.00)	35.84 ^a^ (82.27)	2.21 ^c^ (42.00)	27.67 ^a^ (83.44)
Saturated fat (g)	4.17 ^a^ (6.71)	0.01 ^b^ (0.02)	6.74 ^a^ (15.38)	3.91 ^a^ (5.26)	4.80 ^a^ (5.08)
Potassium (mg)	254.06 ^a^ (676.85)	70.10 ^c^ (74.54)	431.58 ^ab^ (603.43)	612.08 ^b^ (449.50)	402.58 ^ab^ (560.62)
Iron (mg)	3.15 ^a^ (2.95)	0.14 ^c^ (0.24)	1.87 ^ab^ (2.69)	2.42 ^a^ (3.03)	1.56 ^b^ (2.14)
Vitamin D (IU)	3.42 ^a^ (16.67)	0.00 ^b^ (0.00)	5.62 ^a^ (17.71)	3.00 ^a^ (21.75)	2.73 ^a^ (14.91)
Vitamin E (IU)	0.06 ^a^ (0.20)	0.00 ^b^ (0.00)	0.02 ^a^ (0.22)	0.00 ^a^ (0.18)	0.05 ^a^ (0.15)

Comparison of median nutrient values (and IQR) by recipe function. Letters a–d on a line in rows are to denote the statistical significance: Median values in the same row with at least one letter in common are not significantly different. If no letters are present in a row, none of the median values in that row are significantly different. Significance was determined by the Mann–Whitney U test; *p* < 0.05 for significance. The relative differences in nutrient content across recipe functions are shown as the medians for each nutrient. Recipes identified as healthy were the highest in vitamin C (except vs. foods to share; *p* = NS), fiber (except vs. easy-to-make; *p* = NS), and potassium (except vs. comfort foods; *p* < 0.05, and easy-to-make and foods to share; *p* = NS for both) and the lowest in cholesterol (*p* < 0.05). Cocktails were about half the calories per serving as all other recipes and were substantially lower in all nutrients evaluated (*p* < 0.01) except for sugars and vitamin C (*p* = NS).

**Table 3:** Comparison of nutrient content of recipes by quantity of seasonings.

	0	1–2	3–4	5–6	7–8	9–10
Kilocalories	194.22 ^b^ (229.95)	230.75 ^ab^ (222.02)	290.86 ^a^ (270.44)	451.26 ^c^ (363.47)	502.23 ^abcd^ (385.66)	618.56 ^d^ (142.33)
Protein (g)	3.27 ^b^ (7.12)	4.49 ^b^ (7.16)	11.53 ^a^ (11.53)	15.10 ^a^ (21.43)	21.02 ^a^ (21.08)	12.16 ^a^ (4.75)
Carbohydrates (g)	19.87 ^ab^ (38.07)	26.76 ^ab^ (34.00)	30.36 ^ab^ (26.12)	38.93 ^ab^ (40.86)	40.52 ^a^ (42.64)	58.02 ^b^ (20.20)
Fat (g)	5.34 ^c^ (18.94)	9.69 ^bc^ (15.08)	14.25 ^ab^ (17.53)	20.39 ^a^ (32.86)	20.44 ^ad^ (25.70)	40.48 ^d^ (10.21)
Sodium (mg)	174.45 ^b^ (400.49)	297.07 ^c^ (504.17)	754.72 ^a^ (907.27)	770.31 ^a^ (722.18)	773.67 ^ac^ (1579.55)	1876.59 ^d^ (542.82)
Vitamin A (IU)	139.00 ^b^ (424.13)	256.31 ^c^ (572.11)	384.56 ^d^ (697.61)	1894.79 ^a^ (6365.25)	1194.37 ^ad^ (2034.15)	2679.99 ^ad^ (427.10)
Vitamin C (mg)	0.61 ^b^ (7.05)	2.49 ^c^ (12.25)	16.20 ^a^ (22.83)	28.56 ^a^ (31.98)	17.43 ^a^ (40.82)	88.38 ^a^ (18.73)
Calcium (mg)	31.88 ^c^ (74.46)	45.83 ^ac^ (99.05)	67.95 ^a^ (82.95)	150.49 ^bd^ (38.62)	81.29 ^abd^ (205.40)	135.41 ^d^ (35.86)
Fiber (g)	1.22 ^c^ (2.81)	1.11 ^c^ (2.82)	4.25 ^b^ (3.43)	7.64 ^a^ (4.25)	2.80 ^ab^ (3.85)	6.93 ^b^ (0.49)
Sugar (g)	11.32 ^ab^ (19.76)	13.53 ^a^ (16.69)	4.86 ^b^ (7.86)	9.25 ^a^ (18.04)	10.70 ^ab^ (5.74)	33.85 ^a^ (22.48)
Cholesterol (mg)	1.10 ^a^ (35.35)	11.63 ^a^ (63.79)	17.45 ^a^ (48.48)	4.09 ^ab^ (127.58)	39.69 ^ab^ (40.68)	139.50 ^b^ (139.50)
Saturated fat (g)	1.45 ^a^ (7.31)	3.54 ^a^ (7.31)	3.21 ^a^ (3.96)	4.74 ^a^ (6.85)	10.43 ^ab^ (11.63)	5.32 ^b^ (0.26)
Potassium (mg)	126.36 ^b^ (321.72)	121.22 ^b^ (337.89)	427.95 ^c^ (543.76)	866.59 ^a^ (290.04)	627.64 ^c^ (381.24)	799.23 ^ac^ (6.90)
Iron (mg)	0.68 ^b^ (2.69)	1.04 ^b^ (1.46)	1.63 ^c^ (3.17)	4.15 ^a^ (3.19)	3.71 ^ac^ (2.58)	4.29 ^a^ (1.10)
Vitamin D (IU)	0.15 ^a^ (8.01)	2.56 ^a^ (16.28)	0.00 ^a^ (8.51)	8.98 ^ab^ (30.24)	3.01 ^a^ (21.95)	36.73 ^b^ (24.77)
Vitamin E (IU)	0.00 (0.13)	0.03 (0.18)	0.00 (0.09)	0.05 (0.31)	0.00 (0.00)	0.59 (0.59)

Comparison of median nutrient values (and IQR) by the quantity of seasonings. Letters a–d on a line in rows are to denote the statistical significance: Median values in the same row with at least one letter in common are not significantly different. If no letters are present in a row, none of the median values in that row are significantly different. Significance was determined by the Mann–Whitney U test; *p* < 0.05 for significance. The nutrient content (median) per serving compared by the number of seasonings included in each recipe showed the most substantial differences (*p* < 0.001) for protein, sodium, vitamin A, vitamin C, calcium, fiber, potassium, and iron for recipes including 3–6 added seasonings compared with those with none. Comparing 1–2 added seasonings to 5–6 also showed significant differences for the same nutrients (*p* < 0.001). Comparing 3–4 and 5–6 showed significantly higher amounts of fiber, potassium, and iron with more seasonings (*p* < 0.001). Additional seasonings beyond 5–6 did not show further increases in the nutrient content (*p* = NS).

## Data Availability

The data used in this study are available upon request from the corresponding author.
